# Erythrocytes of Little Ground Squirrels Undergo Reversible Oxidative Stress During Arousal From Hibernation

**DOI:** 10.3389/fphys.2021.730657

**Published:** 2021-10-07

**Authors:** Nisred K. Klichkhanov, Elena R. Nikitina, Zainab M. Shihamirova, Maria D. Astaeva, Shamil I. Chalabov, Aleksandr I. Krivchenko

**Affiliations:** ^1^Department of Biochemistry, Dagestan State University, Makhachkala, Russia; ^2^Laboratory of Comparative Physiology of Respiration, Sechenov Institute of Evolutionary Physiology and Biochemistry, Russian Academy of Sciences, St. Petersburg, Russia

**Keywords:** hibernation, arousal, Spermophilus, erythrocytes, oxidative stress, antioxidant defense

## Abstract

The hibernation of small mammals is characterized by long torpor bouts alternating with short periods of arousal. During arousal, due to a significant increase in oxygen consumption, tissue perfusion, and the launch of thermogenesis in cells, a large amount of reactive oxygen species (ROS) and nitrogen (RNS) can be formed, which can trigger oxidative stress in cells. To estimate this possibility, we studied the intensity of free-radical processes in the red blood cells (RBCs) of little ground squirrels (LGS; *Spermophilus pygmaeus*) in the dynamics of arousal from hibernation. We found that in the torpid state, the degree of generation of ROS and RNS (8.3%, *p*>0.09; 20.7%, *p*<0.001, respectively), the degree of oxidative modification of membrane lipids and RBC proteins is at a low level (47%, *p*<0.001; 82.7%, *p*<0.001, respectively) compared to the summer control. At the same time, the activity of superoxide dismutase (SOD) and catalase (CAT) in RBC is significantly reduced (32.8%, *p*<0.001; 22.2%, *p*<0.001, respectively), but not the level of glutathione (GSH). In the torpid state, SOD is activated by exogenous GSH in concentration-dependent manner, which indicates reversible enzyme inhibition. During the arousal of ground squirrels, when the body temperature reaches 25°C, RBCs are exposed oxidative stress. This is confirmed by the maximum increase in the level of uric acid (25.4%, *p*<0.001) in plasma, a marker of oxidative modification of lipids [thiobarbituric acid reactive substances (TBARS); 82%, *p* < 0.001] and proteins (carbonyl groups; 499%, *p* < 0.001) in RBC membranes, as well as the decrease in the level of GSH (19.7%, *p* < 0.001) in erythrocytes relative to the torpid state and activity of SOD and CAT in erythrocytes to values at the Tb 20°C. After full recovery of body temperature, the level of GSH increases, the ratio of SOD/CAT is restored, which significantly reduces the degree of oxidative damage of lipids and proteins of RBC membranes. Thus, the oxidative stress detected at Tb 25°C was transient and physiologically regulated.

## Introduction

Homeothermic animals in winter face a vitally important problem of maintaining temperature homeostasis, which is indispensable for survival. During evolution, some homeothermic animals developed several adaptive strategies that allowed them to endure extreme conditions. One of these strategies is hibernation, which is employed by some mammals to evade such challenges as shorter daylight hours, low ambient temperatures, and scarce food supply in winter time (Ruf and Geiser, 2015; Staples, 2016). During hibernation, physiological functions are significantly suppressed compared to the active summer animals. For example, in the Arctic ground squirrels (*Spermophilus parryii*), oxygen uptake during hibernation drops by more than 90% lowering thereby body temperature (Tb) almost to the ambient level (1–5°C; Tøien et al., 2001; Geiser, 2004). Ground squirrels hibernating at Tb 5°C have a heart rate ranging from 5 to 10 beats per minute (bpm) compared to 350–400bpm in the euthermic state, while the organ perfusion rate decreases more than by 10% vs. the norm (McCarron et al., 2001). The respiratory rate drops from 100–150 to 1–2 breaths per min with prolonged apneic episodes. Blood pressure can drop from 130/80 to 90/30mmHg, and cardiac output – to 1/60 of the euthermic level (Geiser, 2004).

In small rodents, hibernation is discontinuous and consists of alternating torpor-arousal cycles (Heldmaier et al., 2004). The euthermic state that follows arousal persists for several (12–24) hours before the next hibernation bout begins (Carey et al., 2003a). Arousal of animals is accompanied by a rapid reсovery of many physiological functions, including Tb (35–38°C), oxygen uptake, and metabolic rate (Carey et al., 2003a; Muleme et al., 2006). In mammals, fast tissue reperfusion accompanied by increased mitochondrial respiration and oxygen levels during arousal can stimulate the enhanced generation of reactive oxygen species (ROS; Hermes-Lima and Zenteno-Savín, 2002; Turrens, 2003; Sanderson et al., 2013). ROS, including the most biologically significant oxidizing agents, such as O_2_^•−^ and Н_2_О_2_ (Kowaltowski et al., 2009), are generated particularly intensely when mitochondria regain access to O_2_ after a relatively inactive torpid state (Murphy, 2009; Chouchani et al., 2016b). Such a situation is typically observed during ischemia/reperfusion, leading to oxidative damage of tissues (de Groot and Rauen, 2007; Lin and Wang, 2016).

Previously, it was shown that the level of malondialdehyde (MDA), a product of lipid peroxidation (LPO), in the blood plasma and membranes of erythrocytes of the black bear (*Ursus americanus*; Chauhan et al., 2002), as well as the level of conjugated dienes in the intestines of the 13-lined ground squirrels, increased significantly during hibernation (Carey et al., 2000). Recently, Wei et al. (2018) found that in the Daurian ground squirrels (*Spermophilus dauricus*), the Н_2_О_2_ level in the heart and brain and the MDA level in the brain during late torpor, immediately before arousal were higher compared to those in summer active animals, while activity of the antioxidant enzymes such as superoxide dismutase (SOD), catalase (CAT), and glutathione peroxidase (GP) was lower compared to the onset of the torpid state (early torpor) and interbout wakefulness (interbout arousal). These data indicate the presence of oxidative stress in the torpid state. At the same time, Ma et al. (2005) reported no evidence of oxidative damage in the brain of the hibernating Arctic ground squirrels after hibernation, including the absence of cellular stress and inflammatory responses or neuronal pathology. Wei et al. (2018) attempted to find out how ground squirrels manage to avoid the development of oxidative stress during the torpor-arousal cycle. For this purpose, the level of expression of the antioxidant enzymes SOD1, SOD2, CAT, GP, and phosphorylated Nrf2 (pNrf2) in various tissues of Daurian ground squirrels during the summer active period, the early and late stages of the torpid state, and interbout wakefulness were studied. Under oxidative stress conditions, Nrf2 is released from Keap1 and moves to the nucleus (Itoh et al., 1999). It activates transcription factors that increase the expression of gene products controlled by Nrf2, such as Cu/ZnSOD and HO-1 (Bellezza et al., 2018). Wei et al. (2018) found increased p-Nrf2 expression in the heart, brain, kidney, and especially in the liver at the end of the torpid state. The authors hypothesized that ROS generated at the end of the torpid state activate the Nrf2/Keap1 pathway, which triggers the expression of antioxidant enzymes required to protect tissues from oxidative stress during interbout wakefulness and re-entering torpor. It should be noted that these studies were carried out on torpid and awake ground squirrels, and hence it remains obscure how the intensity of free radical processes in tissues changes in the arousal dynamics. In this regard, the results obtained by Tøien et al. (2001) are of interest. The authors analyzed oxygen uptake, plasma concentrations of ascorbic and uric acids, and Tb during arousal of the Arctic ground squirrels. It turned out that during arousal, the maximum rate of decrease in plasma ascorbate coincided with the peak oxygen consumption and peak plasma urate production. These data allowed the authors to assume that at a certain stage of arousal animal tissues develop oxidative stress and that ascorbate can function as an antioxidant during this period. At the same time, there is almost no experimental evidence of free radical processes activation in arousal dynamics of the hibernators. A study of the antioxidant activity of Syrian hamsters (*Mesocricetus auratus*) blood plasma in the early (Tb 14°C) and late (Tb 32°C) stages of awakening showed that the total superoxide radical-scavenging activity of plasma, extracellular SOD activity, and CAT activity reached a maximum at Tb 32°C, which coincided with the peak of uric acid accumulation in plasma – an indicator of ROS generation (Ohta et al., 2006; Okamoto et al., 2006; Akita et al., 2007).

Nitric oxide (NO) is widely considered one of the most important molecules produced in the body, acting as a necessary regulator in a vast array of vital physiological functions, namely, blood pressure, immune response, and neural communication. At the same time, under elevated O_2_^•−^ levels, NO reactivity is shifted toward the formation of peroxynitrite, which can decompose to nitrate or lead to the formation of NO_2_ and hydroxyl radicals. Another feature of the ONOO^−^/NO_2_ pathway is its capacity to generate reactive intermediates that can oxidize thiols to thiol radicals (RS^•^), which may directly react with NO, a process referred to as oxidative nitrosylation. Tissue nitrate/nitrite ratios can serve as indicators of the balance between local oxidative and nitrosative stress (Bryan, 2015). Increased ROS concentrations reduce bioactive NO levels by chemical inactivation. This process is often called NO uptake and is the most significant consequence of oxidative stress (Pierini and Bryan, 2015). It is not known how the NO level in the blood changes during the arousal of ground squirrels. Only it was shown that at the late stage of arousal of golden hamsters, the number of renal and mesenteric arteries containing NO-synthase cells increases relative to the torpid state (Saitongdee et al., 1999). During hibernation, red blood cells (RBCs) must adequately supply oxygen to tissues under conditions of changing Tb. RBCs are potentially sensitive to damaging effects of ROS produced both in the cells themselves and in the blood plasma (Pandey and Rizvi, 2011; Kuhn et al., 2017). Exactly for this reason, RBCs have an effective system of antioxidant protection which includes glutathione (GSH), vitamins C and E, enzymes (SOD, CAT, GP, glutathione S-transferase, and glutathione reductase), and thioredoxins (Çimen, 2008). Regulating the oxygen affinity of hemoglobin can also play an important role in preventing oxidative stress during hibernation. It has been shown that in hibernating mammals the oxygen affinity of hemoglobin increases, decreasing thereby oxygen release in tissues due to their low metabolic activity (Revsbech and Fago, 2017). During awakening from hibernation, an increase Tb and 2,3-DPH level (Tempel and Musacchia, 1975) can reduce the oxygen affinity of hemoglobin and thus promote rapid tissue oxygenation and stimulate ROS production.

In this study, we hypothesized that RBCs of the little ground squirrels (LGS) are exposed to oxidative stress during arousal from hibernation but can regulate their antioxidant defense to prevent or minimize potential oxidative damage to RBCs during the torpor-arousal cycle. We aimed to find out how the degree of oxidative modification of lipids and proteins in RBC membranes changes and how the antioxidant system of RBCs changes during the arousal of ground squirrels from hibernation. We established that in the arousal dynamics oxidative stress develops in RBCs at Tb 25°C. However, after the completion of arousal, the total antioxidant capacity of RBCs increases and oxidative stress disappears. Therefore, oxidative stress that arises in RBCs during arousal is controllable.

## Materials and Methods

### Animals

**Figure 1:**
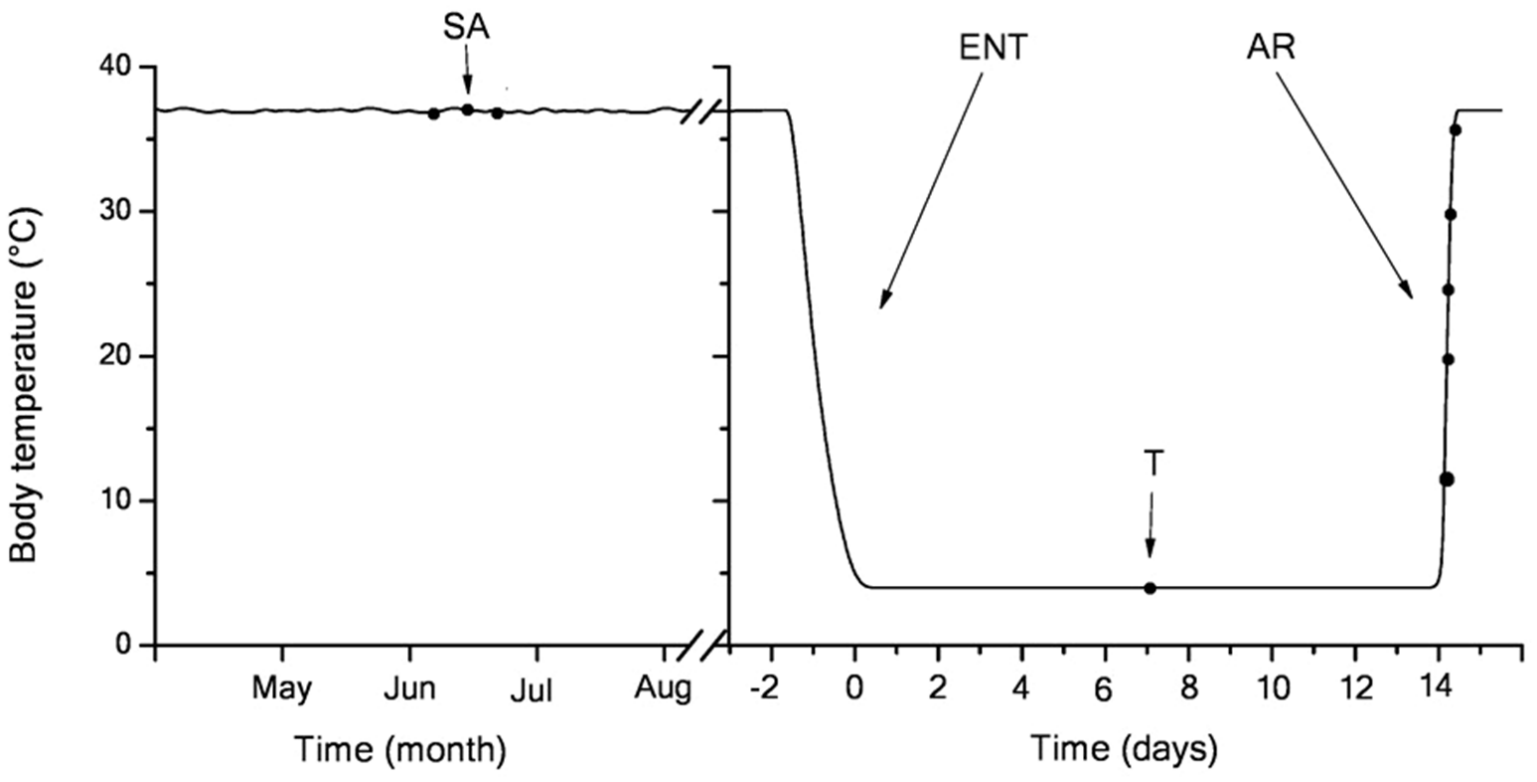
Body temperature dynamics in the hibernating little ground squirrels (LGS; *y*-axis) vs. the month of the year (left; three-letter code; starting from May) and days of a single bout at the end of January (right; *x*-axis). Blood samples were retrieved at seven time points (black dots) coinciding with summer activity (left) or hibernation (right): in mid-torpor and during arousal (Tb 4–37°C). After entering torpor, Tb slowly decreased from euthermic (36±1°C) to near-ambient temperature (3.5–4.5°C). During 2-week torpor, Tb was maintained within 3.8±1°C. Body temperature in summer active and torpid animals was measured every day, upon entering torpor − every hour, during arousal − continuously. During arousal, animals rapidly rewarm to euthermic Tb levels. SA, summer active; ENT, entry to torpor; T, mid-torpor; and AR, arousal.

The studies were carried out on the LGS, which are typical heterothermic mammalian hibernators. Adult LGS of both sexes were trapped during May in the foothills of the Daghestan Republic (Russia). LGS were housed individually at 23–25°C and fed a rodent chow diet, sunflower seeds, fresh carrots, and apples until mid-October, when they were transferred to a cold chamber set to an ambient temperature of 3–5°C (a typical resident temperature in burrow for LGS) a 4:20h light-dark cycle. Several days later, all animals fell into hibernation, with the first torpor bout lasting 4–5days. The duration of individual torpor bouts was determined by a sawdust technique. Briefly, this method implies applying a small amount of sawdust over the back of a torpid LGS followed by daily observations of whether the sawdust is still in place. Rectal Tb was measured immediately before euthanasia using the MS6501 thermometer (Mastech, Hong Kong). All treatment procedures of LGS were carried out according to the Animal Welfare act and the University Guide for Care and Use of Animals. The hibernation experiments were carried out from January through February when the bout duration was 14–15days. A total of 42 weight-matched LGS were randomly combined into seven groups (*n*=6 in each group). The female to male ratio was usually 3:2 per group. In experimental hibernating animals, samples were retrieved in the following states (Figure 1): (1) summer active group sampled in June–July: Tb was 36–38°C; (2) torpor group: after 2-month hibernation (61+8days), animals entered a new torpor bout with Tb maintained at 3.5–4.5°C for 6–7days (mid-bout); and (3–7) arousing group: after 2-month hibernation, the bout duration was 14–15days; in the middle of torpor bouts (6–7days) animals that had gone through the torpor-arousal cycle more than 8–9 times were transferred in to the room with a temperature of 25°C for arousal. LGS were involved in experiments when the rectal temperature reached 10, 20, 25, 30, and 37°С. The animals were anesthetized by intraperitoneal injection with sodium pentobarbital (50mg/kg, intraperitoneally) and sacrificed by decapitation. Torpid animals were killed by cervical dislocation (to prevent initiation of arousal).

### Blood Sampling

Immediately after decapitation, blood was collected into heparin tubes (50U/ml). After centrifugation at 3,000*g* for 10min at 4°C, the plasma and buffy coat were removed. Erythrocytes were purified by 3-fold resuspending and washing with 20 volumes of phosphate-buffered saline (PBS) containing 1mM ethylenediaminetetraacetate (EDTA) and 0.5mM phenylmethylsulfonyl fluoride. The plasma was used to measure uric acid, nitrate/nitrite, and thiobarbituric acid reactive substances (TBARS). Packed RBCs were used for TBARS assay. The level of protein carbonyls was determined in RBC membranes. To estimate SOD, CAT, and reduced glutathione (GSH), washed RBCs were hemolysed using bidistilled water (1:9v/v; for 10min), centrifuged at 10,000*g* for 15min, and the supernatant was removed to be immediately analyzed.

### Preparation of Erythrocyte Membranes

Red blood cell ghosts were prepared from washed cells according to the method of Dodge et al. (1963). RBCs were hemolysed on ice with 20 volumes of 10mM phosphate buffer (pH 7.4) containing 1mM EDTA and 0.5mM PMSF, and centrifuged at 20,000*g* for 20min. Ghosts were resuspended in ice-cold 5mM phosphate buffer (pH 7.4) and recentrifuged under the same conditions until deprived of residual hemoglobin. White ghosts were stored at −70°C until use.

### Determination of Plasma Uric Acid Level

The plasma uric acid level was assessed using the Olvex Diagnosticum assay kits (Russia) according to the manufacturer’s protocol. This determination is based on conjugate reactions catalyzed by uricase and peroxidase. Uricase acts on uric acid to produce allantoin, carbon dioxide, and hydrogen peroxide. Hydrogen peroxide in the presence of peroxidase reacts with a chromogen (4-aminoantipyrine and dichlorophenol sulphonate) to yield quinoneimine, a red-colored complex. The absorbance measured at 505nm is proportional to the amount of uric acid in the specimen.

### Determination of Plasma NO Level

Plasma levels of NOx (nitrite+nitrate), which is a stable end products of nitric oxide, were measured by the total amount of nitrites, the level of which was determined by the color reaction with the Griss reagent after nitrate reduction by activated cadmium (Navarro-Gonzálvez et al., 1998). Three hundred microliter of the plasma were deproteinized by adding 250μl of 75mmol/L ZnSO_4_. Samples were stirred and centrifuged at 10,000*g* for 5min with 350μl of 55mmol/L NaOH added thereafter. The solution was stirred again and centrifuged at 10,000*g* for 5min. Then, 750μl of the resulting supernatant was mixed with 250μl of glycine buffer (45g/L, pH 9.7). Cadmium granules were washed three times with deionized water and mixed with 5mmol/L CuSO_4_ solution in glycine-NaOH buffer (15g/L, pH 9.7) for 5min. Copper-coated granules were used within 10min. Freshly activated cadmium granules (2g) were added to 1ml of pretreated deproteinized plasma or calibrator (sodium nitrate was used as a standard). After stirring for 10min, the samples were transferred into tubes for the determination of nitrites. To evaluate the total amount of the nitrite ion, 375μl of N-naphthylethylenediamine (0.02g per 100ml of distilled water) and 400μl sulfanilic acid (1g per 100ml of 3mol/L HCl) were added to 100μl of the sample. The optical density of the samples was measured at a wavelength of 540nm. The nitrate+nitrite ion (NOx) level was expressed in μmol per L of plasma.

### TBARS Assay in Plasma and Red Blood Cells

Lipid peroxidation was assayed by measuring TBARS, which primarily measures MDA generation (Rice-Evans et al., 1991). One millilitre of plasma or washed RBCs were mixed with 1ml 20% trichloroacetic acid (TCA), 1ml 2% butylated hydroxytoluene, and 1ml 0.37% 2-thiobarbituric acid in 0.25M HCl. The samples were heated to 100°C for 10min and centrifuged at 600*g* for 10min (20°C). The supernatant absorbance was measured at 532nm vs. the blank. The TBARS level was calculated using an extinction coefficient for MDA, *ɛ*=1.56×10^5^M^−1^ cm^−1^, and expressed in μmoles per L of plasma or erythrocytes.

### Protein Carbonyl Assay

Protein carbonyl content in RBC membranes was measured according to the method described by Venditti et al. (2004). Briefly, 0.2ml of membrane suspension (2.5mg protein per mL) were mixed with 0.2ml 20% TCA and centrifuged at 1,500*g* for 10min. For each determination, three TCA-precipitated samples containing 0.5mg of protein were dissolved in 100μl of 0.1M NaOH for 5min. Then, 1ml of 10mM 2,4-dinitrophenylhydrazine (DNPH) solution in 2N HCl was added, and the mixture was incubated for 1h at room temperature while shaking intermittently. The control sample was added with 2N HCl only. Then, 20% TCA (w/v) was added to both tubes and the mixture was incubated for 10min on an ice bath. The tubes were then centrifuged at 1,500*g* for 15min to obtain protein pellets. Protein pellets in all the tubes were washed three times with a washing solution (ethanol:ethyl acetate, 1:1, v/v) to remove unreacted DNPH and remnant lipids. Finally, pellets were dissolved in 6 M guanidine hydrochloride and incubated for 10min at 37°C. The absorbance of the resulting protein solution was recorded at 370nm. The difference in the sample vs. control optical density was determined and expressed in nmol of carbonyl groups/mg of protein using a molar extinction coefficient of 22,000M^−1^ cm^−1^. The protein content of RBC membranes was estimated by Lowry et al. (1951).

### Determination of GSH Level in Erythrocytes

The GSH concentration in RBC was determined by a modified Elman method (Ansari et al., 2008). Around 0.2ml of a 20% sulfosalicylic acid solution were added to 0.6ml of RBC hemolysate (1 volume of RBC+9 volumes of distilled water). Samples are mixed and centrifuged at 2,000*g* for 10min at 2°C. Around 0.2ml of supernatant were transferred to tubes containing 2.55ml of 0.1M Tris buffer (pH 8.0) with 0.01% EDTA. The resulting mixture was added with 0.025ml of 0.4% of 5,5'-Dithiobis-(2-nitrobenzoic acid) solution in methanol. The intensity of the resulting yellow color was immediately measured at 412nm on a spectrophotometer. The GSH concentration was calculated using the 5-thio-2-nitrobenzoic acid extinction coefficient (13.6mmol^−1^ Lcm^−1^) and expressed in mmol per L of erythrocytes.

### Determination of SOD and CAT Activity

Cu/Zn-SOD activity was measured as in Fried (1975). To remove hemoglobin, hemolysates for assaying SOD activities were prepared according to DiSilvestro (1989). Briefly, 1ml of hemolysate was treated with 0.3ml of chloroform and 0.15ml of ethanol, mixed thoroughly for 30min at 4°C, and precipitated hemoglobin was removed by centrifugation at 10,000*g* for 15min. The supernatant was removed and used to assay SOD activity. To do this, 0.05ml of RBC lysate was added to the reaction mixture (2.85ml) containing 100mM phosphate buffer (pH 7.8), 27μM EDTA, 4mM Nitroblue tetrazolium, and 65μM phenazine methosulfate. The reaction was triggered by adding 0.1ml of 1mM NADH. Samples were incubated at 37°C for 10min in the dark. The optical density of the samples was measured on a spectrophotometer at 540nm vs. control containing all the components of the incubation medium except NADH. The percentage inhibition of Nitro blue tetrazolium reduction was a measure of the SOD activity. The amount of the enzyme that caused 50% inhibition of Nitro blue tetrazolium reduction was taken as one unit (IU). SOD activity was expressed as U per mg Hb. Hemoglobin was assayed by a cyanmethemoglobin method of Crosby et al. (1954).

#### Temporal Kinetics of SOD Activation by GSH

Red blood cell lysate purified from hemoglobin (obtained from hemolysate with a concentration of 3.5mg Hb/ml) was incubated with 100μM GSH for 10, 20, 30, 40, 60, and 80min at 37°C, diluted 40 times with 0.1M phosphate buffer (pH 7.8), and SOD activity was then assayed.

#### Measurement of SOD Activation by GSH

Hemoglobin-free hemolysate obtained from hemolysate with a concentration of 3.5mg Hb/ml was incubated with various concentrations of GSH ranging from 25 to 200 μM for 40min at 37°C, diluted with 0.1M phosphate buffer, pH 7.8, and then SOD activity was assayed.

*Catalase activity* was measured in hemolysates at 37°C on the Beckman coulter Du 730 spectrophotometer according to the Aebi method (Aebi, 1974). RBC lysate (0.03mg Hb) was added to the reaction mixture (1ml) containing 100mM sodium phosphate buffer (pH 7.4) and 8.8mM H_2_O_2_. The absorbance decrement at 240nm was monitored for 3min at 37°C. The amount of CAT capable of transforming 1.0μmol of H_2_O_2_ for a minute was taken as an enzyme activity unit (IU). CAT activity was expressed in IU per mg Hb.

### Statistical Analysis

Statistical analyses were performed using one-way ANOVA and Tukey’s test (*p*<0.001). For correlation between uric acid, NOx, TBARS, protein carbonyl groups, GSH, SOD, and CAT, we used Spearman’s test (*p*<0.001). The significance level *p*<0.001 was used due to multiple endpoints. Calculations were performed using R Core (version 3.4.0).

## Results

### In Torpor, the Blood Shows a Low Level of Free Radical Processes

**Figure 2:**
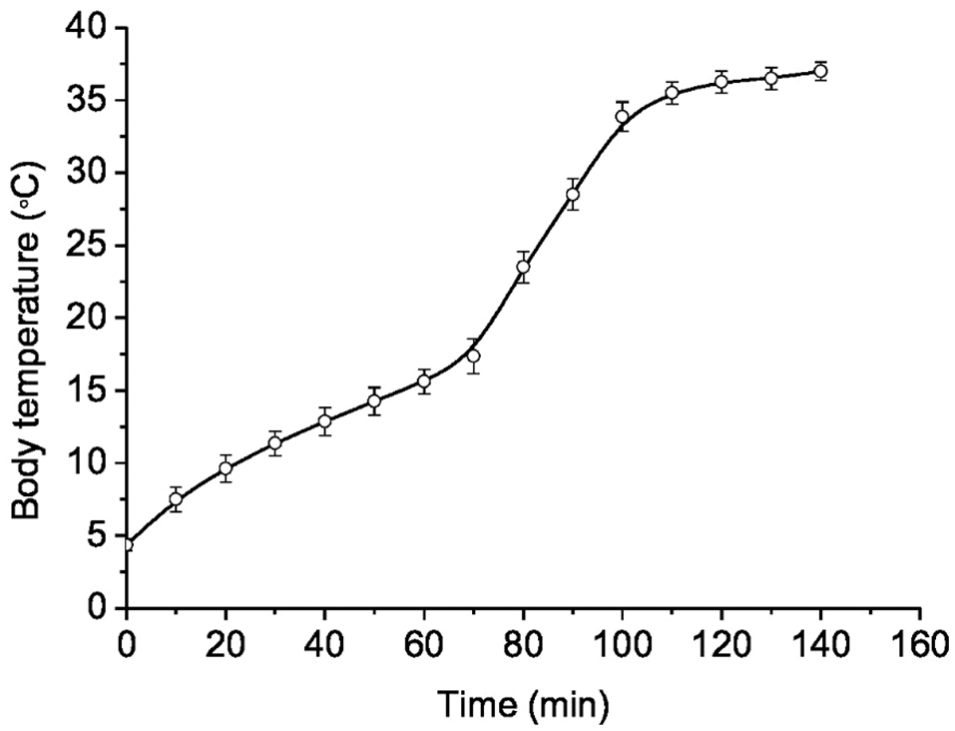
Dynamics of body temperature recovery during arousal from hibernation (Tb~4°C) in ground squirrels transferred to a room with a temperature of 25°C (*n*=12). Abscissa – heating time (min); ordinate – body temperature (°C). Data are presented as mean±SEM.

In torpid LGS, the Tb was 3.8±0.4°C. The transfer of animals from individual cages to a room with a temperature of 25°C contributed to their arousal. A rise in Tb up to 37°C and their complete arousal took 2.2±0.2h (Figure 2). The rewarming rate (WR) during arousal was different. At the beginning of arousal, when Tb increased from 4 to 10°C due to passive warming, the WR was 0.25°C/min, with subsequent self-warming occurring at WR of 0.14°C/min; within the Tb range of 17–35°C, the WR increased to 0.51°C/min. The lowest WR (~0.07°C/min) was found in the Tb range of 35–37°C. Strong contractions of the chest muscle and explosive tremors that became visually noticeable in the Tb range of 17–35°C promoted a sharp increase in Tb. These results are consistent with data by Lee et al. (2002) obtained on hibernating bats (*Rhinolophus ferrumequinum*).

**Figure 3:**
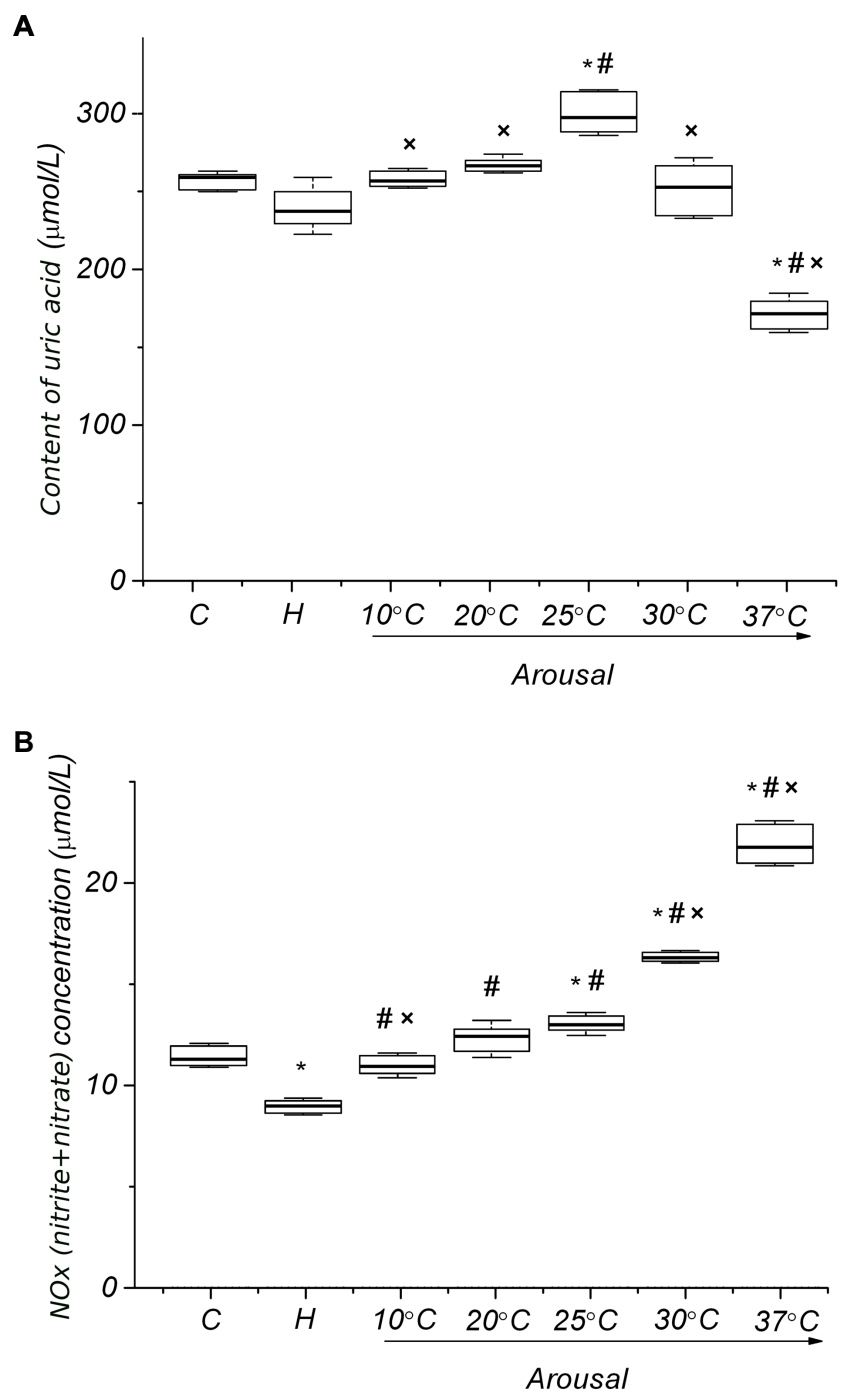
Plasma levels of uric acid **(A)** and NOx **(B)** of ground squirrels during arousal from hibernation. C, control; H, hibernation; and body temperature (°C; *n*=6). Data are presented as box plots that show the median, 25/75 percentiles (box), and 10/90 percentiles (bars). ^*^p < 0.001, compared with C; ^#^*p* < 0.001, compared with H; ^×^*p* < 0.001, compared with Tb 25°C.

Many researchers report that in the torpid state, tissue level of reactive oxygen (ROS; Tøien et al., 2001; Yin et al., 2016) and nitrogen species (NOS; Henning et al., 2002; Sandovici et al., 2004) decrease. Our data on plasma levels of uric acid (Figure 3A) and NOx (Figure 3B) in hibernating ground squirrels are consistent with these results.

In the torpid state, the level of uric acid and NOx in plasma is lower compared to active animals in summer (8.3%, *p*>0.09; 20.7%, *p*<0.001, respectively).

**Figure 4:**
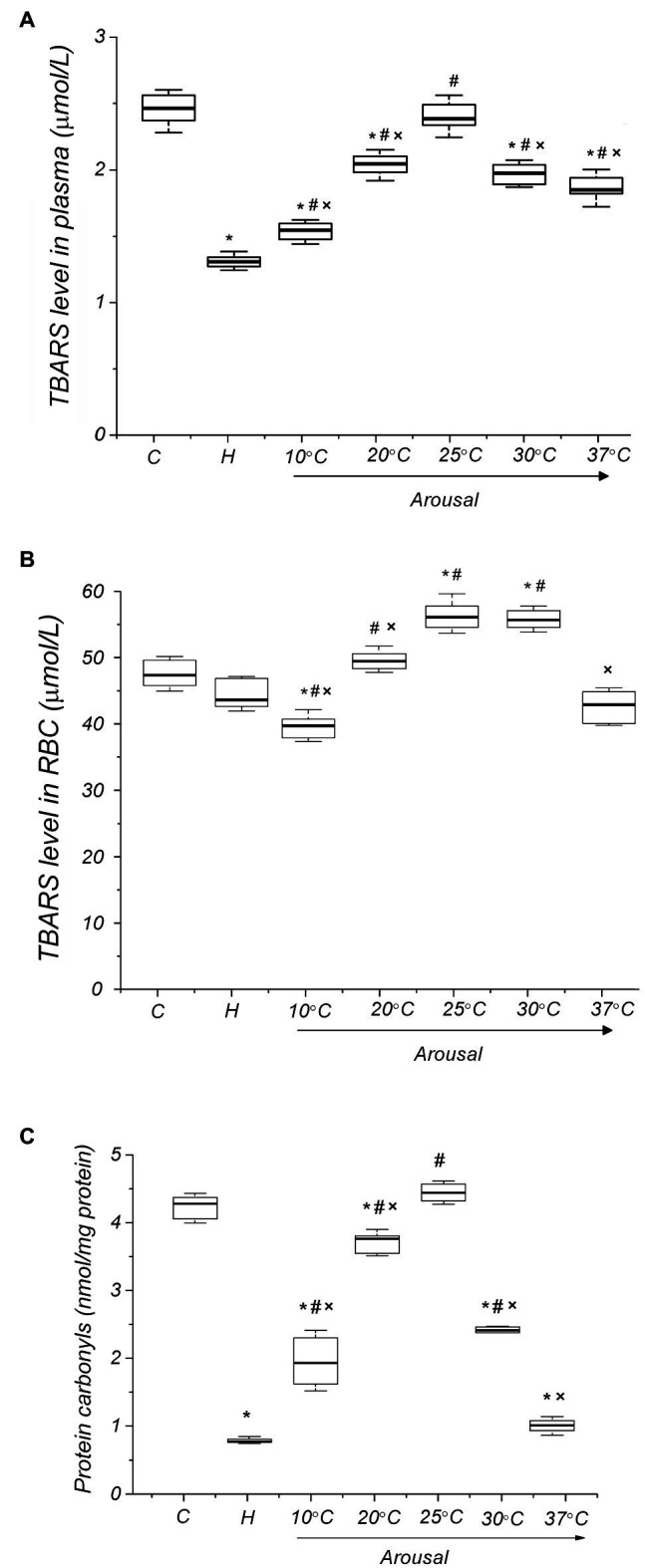
Level of oxidative modification products of lipids and proteins of blood plasma and RBC membranes in ground squirrels during arousal from hibernation. Plasma TBARS dynamics (panel **A**), TBARS in RBCs (panel **B**) and carbonyl groups in RBC membrane proteins (panel **C**) during torpor and arousal (*n*=6). C, control; H, hibernation; and body temperature (°C). Data are presented as box plots that show the median, 25/75 percentiles (box), and 10/90 percentiles (bars). ^*^p < 0.001, compared with C; ^#^*p* < 0.001, compared with H; ^×^*p* < 0.001, compared with Tb 25°C.

In order to estimate the level of ROS-induced oxidation of lipids and proteins in the torpid state, we determined plasma and RBC levels of TBARS and protein carbonyls. The plasma TBARS level was significantly lower (47%, *p* < 0.001) in torpid animals compared to the control (Figure 4A). The RBC level of TBARS was also reduced but less than in plasma (7.7%, *р* ˃ 0.1; Figure 4B). In torpid LGS, the level of carbonyl groups in RBC membrane proteins was significantly (5.8 times) lower [0.74 (0.72, 0.82) nmol/mg, *р* < 0.001] compared to control animals [4.27 (3.98, 4.42) nmol/mg; Figure 4C].

**Figure 5:**
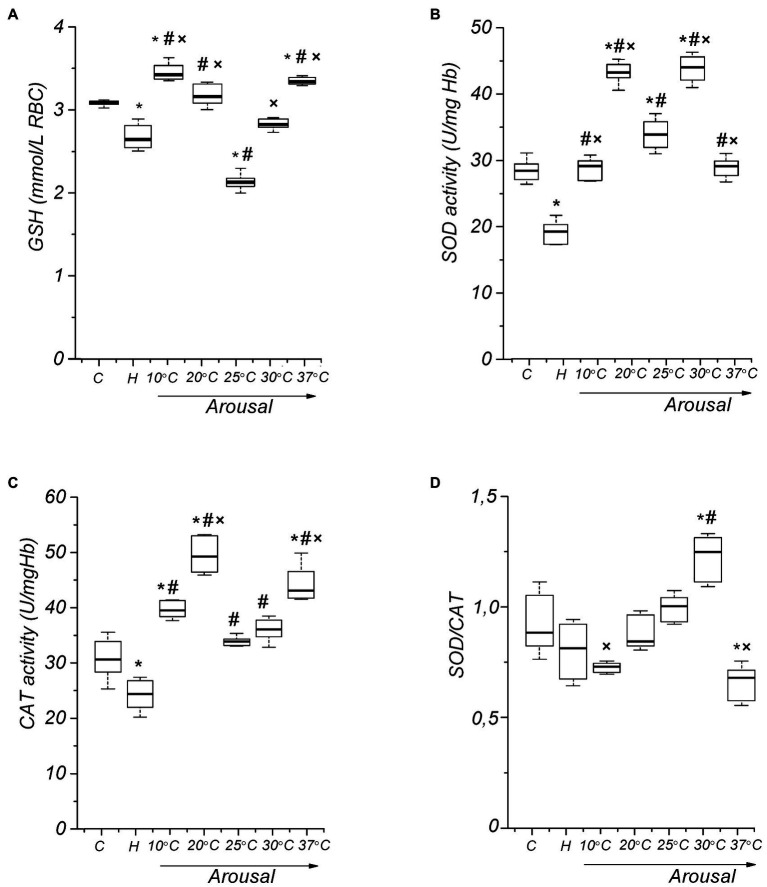
Activity of RBC antioxidant defense components in ground squirrels during arousal from hibernation. Glutathione (GSH) level dynamics **(A)**, superoxide dismutase (SOD) activity **(B)**, catalase (CAT) activity **(C)**, and RBC SOD/CAT ratio **(D)** during torpor and arousal (*n*=14). C, control; H, hibernation; and body temperature (°C). Data are presented as box plots that show the median, 25/75 percentiles (box), and 10/90 percentiles (bars). ^*^p < 0.001, compared with C; ^#^*p* < 0.001, compared with H; ^×^*p* < 0.001, compared with Tb 25°C.

Despite a decreased level of markers of oxidative modification of lipids and proteins in RBC, a small but reliable decrease (13.3%, *p* < 0.001) in GSH level was found in torpid animals compared to the controls (Figure 5A). In the torpid state, the activity of SOD (32.8%, *p*<0.001) and CAT (22.2%, *p*<0.001) in RBCs were significantly reduced compared with control (Figures 5B,C), which is consistent with a decrease in the production of ROS and NOS, as well as in the level of LPO and protein oxidation in RBCs. Such a reduction may have a regulatory character. Usually, in active animals, two key antioxidant enzymes of RBCs, SOD, and CAT, work simultaneously. They also work simultaneously during hibernation, since the ratio of SOD to CAT does not change significantly (8%, *p* > 0.1) compared to active summer animals (Figure 5D).

**Figure 6:**
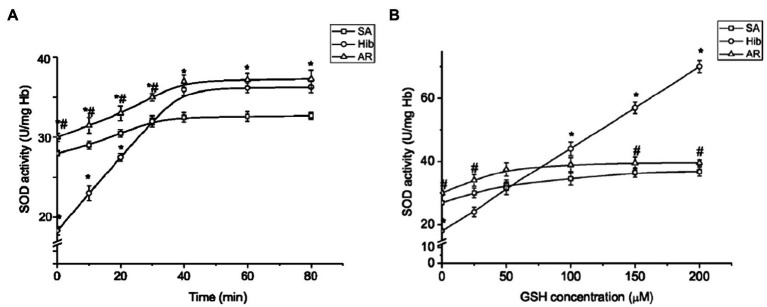
Glutathione promotes SOD activation in ground squirrel RBCs in the torpid state. Temporal kinetics of SOD activation by exogenous GSH in hemolysate (*n*=10; **A**). The effect of GSH on RBC SOD activity (*n*=10; **B**). SA, summer active; HIB, hibernating; and AR, aroused. Data are expressed as mean±SEM; ^*^p < 0.05 vs. SA; ^#^*p* < 0.01 vs. HIB.

A regulatory change in SOD activity has been assumed to occur through the redox state modification of the enzyme’s thiol group (Banks and Andersen, 2019). In addition, the important role of GSH in the activation of SOD has been shown (Carroll et al., 2004). We tested how GSH affects the SOD activity of ground squirrel RBCs by incubating hemolysates with GSH (100μM). Figure 6A shows the data on the dependence of RBC SOD activity on the time of incubation with GSH. It can be seen that in all the physiological states of animals SOD activity increases over time, reaching a maximum after 40min, and then stops changing any longer. It should be noted that in the torpid state SOD activity increases more sharply in the presence of GSH. Next, we investigated the effect of different concentrations of GSH on SOD activity. At the beginning of the studies on summer active ground squirrels, GSH concentrations in the range of 10–500mmol were used to analyze the effect of GSH on the activity of SOD. It turned out that concentrations below 20mmol do not affect the activity of the enzyme. At 300mmol, the effect was the same as at 200mmol, and at 500mmol, the activity of SOD was slightly lower than at 200mmol (data are not given). Based on these data, we focused on the range of GSH concentrations of 25–200mmol in further studies. We found differences in the effects of GSH on SOD activity when changing the physiological state of ground squirrels. An analysis of the dependence of SOD activity on the GSH level shows (Figure 6B) that in the torpid state, in contrast to the control animals and those arousing from hibernation, SOD activity increases linearly in the 25–200μM range of GSH concentrations.

### During Arousal, Red Blood Cells Experience Reversible Oxidative Stress

Orr et al. (2009) demonstrated that increased thermogenesis associated with the arousal of the Arctic ground squirrels leads to tissue-specific oxidative stress in brown adipose tissue (bat) but not in the liver. Whether oxidative stress develops in RBCs during the arousal of ground squirrels from hibernation remained unknown. To clarify this issue, we determined the intensity of ROS and reactive nitrogen species (RNS) generation in the plasma and RBCs at different stages of ground squirrel arousal by the level of uric acid and NOx, TBARS, and protein oxidation. In addition, we studied the level of GSH and the activities of SOD and CAT (the key antioxidant enzymes in RBCs).

During the arousal of ground squirrels, the uric acid level increases, reaching a maximum at Tb 25°C in comparison both to the torpid state (25.4%, *p*<0.001) and control (14.9%, *p*<0.001) (Figure 3A). Further rewarming is accompanied by a decrease in the plasma level of uric acid, and at Tb 37°C, its level significantly decreases compared both to the torpid state (27.9%, *р*<0.001) and control (33.9%, *р*<0.001). An increase in body temperature during arousal leads to an increase in the plasma level of NO metabolites (Figure 3B). A sharp increase in the NOx level was detected in the Tb range of 25–37°C. After complete self-warming of the animals (Tb 37°C), the plasma NOx level was 2.5 times higher [21.83 (20.97, 22.73) μmol/L, *р* < 0.001] than that in torpid animals [8.90 (8.57, 9.13) μmol/L]. No correlation was found between the level of uric acid and NOx for all analyzing groups. During arousal, the plasma TBARS level increased, reaching a maximum at Tb 25°C [2.39 (2.35, 2.41) μmol/L, 82%, *р* < 0.001] compared to the torpid state [1.31 (1.29, 1.32) μmol/L; Figure 4A]. A further rise in Tb leads to a decrease in plasma TBARS levels. After recovery of Tb (37°C), the plasma TBARS level drops by 24.7% (*p* < 0.001) vs. control values. RBCs, both in a torpid state and during rewarming, demonstrate approximately the same dynamics of the TBARS level (Figure 4B) as in the blood plasma. Thus, a decrease in the level of TBARS at the initial stage of warming at Tb 10°C (16%, *p* < 0.001) compared to the control, a significant increase at Tb 25°C compared to the torpid state (28.0%, *p* < 0.001) and control (18.6%, *p* < 0.001) and a decrease to the control values at Tb 37°C. A correlation between plasma and RBC TBARS levels was significant (*r*=0.62, *p*<0.001). During rewarming within the Tb range of 4–25°C, there was a linear increase in the level of protein carbonyl groups in the RBC membranes (Figure 4C). At Tb 25°C, the level of protein carbonyl groups in the RBC membrane reached a maximum [4.43 (4.33, 4.54) nmol/mg, *р* < 0.001], exceeding their level in torpid animals by 5.7 times [0.74 (0.73, 0.77) nmol/mg]. Further rewarming of ground squirrels led to a decrease in the level of carbonyl groups in membrane proteins. When Tb reached 37°C, the level of carbonyl groups in RBC membrane proteins was 4.3 times lower [0.98 (0.91, 1.04) nmol/mg, *р* < 0.001] compared to the control level. A positive correlation was found between the level of carbonyl groups in erythrocyte membrane proteins and the level of uric acid in plasma (*r*=0.71; *p*<0.001) and TBARS in plasma (*r*=0.88; *p*<0.001). A positive correlation was also found between TBARS level in RBCs and the carbonyl groups of erythrocyte membrane proteins (*r*=0.61; *p*<0.001).

Glutathione is the most important low molecular weight antioxidant synthesized in cells (Galano and Alvarez-Idaboy, 2011; Aquilano et al., 2014). It plays an essential role in ROS removal. Therefore, we measured the level of GSH in erythrocytes during the arousal of ground squirrels. Raising Tb to 10°C leads to the restoration of the GSH level in erythrocytes to control values (Figure 5A). However, after that the GSH level decreases, reaching a minimum at Tb 25°С in comparison both to the torpid state (19.7%, *p*<0.001) and control (31%, *p*<0.001). A further increase in body temperature contributes to an increase in the RBC GSH level. At Tb 37°C, the GSH level in RBCs [3.34 (3.31, 3.38) mmol/L, *p*<0.001] slightly exceeds the control values [3.08 (3.07, 3.10) mmol/L]. A negative correlation was found between TBARS and GSH in erythrocytes (*r*=−0.72, *p*<0.001).

To investigate antioxidant defenses, RBCs SOD and CAT activities during the arousal of the LGS were determined. At the onset of rewarming (Tb 10°C), there was a significant increase in RBC SOD activity (45%, *р* < 0.001) compared to that in the torpid state (Figure 5B). A greatest increase in SOD activity compared to control (52%, *р* < 0.001) and the torpid state (126%, *p*<0.001) was observed in animals with Tb 20°C. At Tb 25°C, the SOD activity decreases compared to Tb 20°C (21.9%, *р* < 0.001), but at Tb 30°C it again increased significantly compared both to the torpid state (120.4%, *р* < 0.001), and control (55.2%, *р*<0.001). After complete rewarming, SOD activity decreased to the control level. The activity of SOD significantly and positively correlated with the level of uric acid (*r*=0.48, *p*<0.001), NOx (*r*=0.51, *p*<0.001), TBARS in plasma (*r*=0.48, *p*<0.001), TBARS in RBC (*r*=0.66, *p*<0.001), and carbonyl groups of erythrocyte membrane proteins (*r*=0.50, *p*<0.001).

Catalase activity in the Tb range of 10–25°C changed similarly to SOD, reaching a maximum at Tb 20°C both relative to the torpid state (102.8%, *p* < 0.001) and control (60.9%, *р* < 0.001; Figure 5C). Аfter the complete arousal CAT activity significantly exceeded the control level (in contrast to SOD; 43%, *p*<0.001). During arousal, the SOD/CAT ratio increased, reaching a maximum at Tb 30°C compared to torpor (53.7%, *p*<0.001) and control (41.5%, *p*<0.001), and then (Tb 37°C) significantly decreased compared to the Tb 30°C (45.8%, *p*<0.001) and control (23.3%, *p*<0.001; Figure 5D). CAT activity was significantly and positively correlated with the level of NOx (*r*=0.52, *p*<0.001), GSH (*r*=0.61, *p*<0.001), and SOD activity (*r*=0.48, *p*<0.001).

## Discussion

In this work, we tested how free radical processes are regulated in RBCs of the LGS *Spermophilus pygmaeus* during arousal from hibernation. For this purpose, we evaluated the intensity of ROS and RNS production, the degree of oxidative destruction of lipids and membrane proteins, as well as the activity of the of the antioxidant defense components in RBCs during the summer (when animals are active), hibernation (when animals are torpid), and in the dynamics of arousal. As a result of this work, we have discovered the remarkable capability of RBCs to manage oxidative stress during arousal from hibernation.

During deep torpor, the activity of such key antioxidant enzymes of RBCs as SOD and CAT turned out to be significantly reduced. In the torpid state, we also revealed a slight decrease in the RBC GSH level. This decrease does not appear to be associated with the development of oxidative stress, since levels of the markers both of LPO (TBARS) and oxidative modification of RBC membrane proteins (carbonyl groups) are significantly reduced. A decrease in GSH level was found in the liver, brown adipose tissue, kidneys, and spleen of the Arctic ground squirrel *Spermophilus parryii* (Ma et al., 2004; Orr et al., 2009). It should be noted that Carey et al. (2003b) found a decrease in GR activity in the intestines of hibernating 13-lined ground squirrels. GSH synthesis depends on the activity of the appropriate enzymes and the uptake of precursor amino acids (Meister, 1991). It can be assumed that a drop in the RBC GSH level in the torpid state is associated with a decrease in its biosynthesis due to insufficient supply of precursor amino acids (caused, in turn, by a temperature dependence of enzymes and transporters) and energy deficiency. Doherty et al. (1993) showed that in RBCs of 13-lined ground squirrels the ATP level in the torpid state is about 50% of that in euthermic animals.

A decrease in SOD and CAT activities in RBCs of torpid animals is regulated probably by a decrease in the intracellular level of ROS (as evidenced by low levels of markers of oxidative modification of lipids and RBC proteins). As our data show, in torpid animals, as opposed to those active in the summertime (control) and after complete arousal, RBC SOD is significantly activated in the presence of exogenous GSH (Figure 6). Therefore, the thiol group, which is crucial for SOD functioning, was either oxidized or involved in the formation of mixed disulfides. The Cu/Zn-SOD molecule in human RBCs is a homodimer, with each monomer containing four cysteine residues: C6, C57, C111, and C146. Cysteine residues at positions C57 and C146 are involved in the formation of an intramonomeric disulfide bond responsible for protein stability (Tainer et al., 1983). Cysteine residues at positions C6 and C111 remain vacant. The C111 residue, in contrast to C6, is exposed on the protein surface near the interdimeric interface and can be oxidized or modified (Wilcox et al., 2009). It has been demonstrated that Cu/Zn-SOD isolated from human RBCs can be glutathionylated at C111 (Wilcox et al., 2009). It should be noted that mouse and rat Cu/Zn-SOD have only three cysteine residues (equivalent to positions C6, C57 and C146 in human Cu/Zn-SOD; Chen et al., 2012). In the 13-lined ground squirrel, it also has three cysteine residues (C7, C56, and C145) but no cysteine at position C111. It can be assumed that SOD in LGS RBCs also has three cysteine residues. As judged from the concentration-dependent increase in the enzyme activity in the presence of exogenous GSH, it can be argued that a part of the SOD molecules in the torpid state was glutathionylated and that probably this process involved cysteine at position C7, which could be accessible upon changes in the enzyme conformation (Ghosh et al., 2013). Carroll et al. (2004) showed that GSH activates human SOD1 expressed in yeast or mammalian cells. The authors believe that GSH forms a complex with Cu (I) and then transfers copper ions to the SOD molecule, activating it. It is also possible that GSH can provide the reducing equivalents required to activate Cu in the SOD molecule.

As noted above, ground squirrels are periodically aroused from hibernation, during which Tb quickly rises from near-zero to euthermic levels (Figure 1). In addition to passive warming, brown adipose tissue (BAT) plays an important role in the increase in body temperature during the initial period of arousal, the temperature of which markedly increases yet before the same occurs with the Tb (Horwitz et al., 1968). A significant contribution to further increasing the Tb is made by shivering thermogenesis (ST) which will start when the Tb reaches 15–16°C (Hashimoto et al., 2002; Dark, 2005). In our studies, the highest rate of rewarming was found in the Tb range of 17–32°C due to onset of ST. These results are consistent with the data obtained by Lee et al. (2002) on hibernating bats (*Rhinolopus ferrumequinum*). It should be noted that during the arousal of bats, a rapid increase in Tb well correlated with a significant increase in the oxygen uptake rate (from nearly zero to 11.9ml O_2_/kg/h). Some data indicate that activation of non-shivering thermogenesis (NST) and ST can promote ROS production in tissues. Acute activation of BAT, as well as adrenergic stimulation of adipose cells, increase superoxide production in mitochondria simultaneously with respiration uncoupling that involves the uncoupling protein 1 (Chouchani et al., 2016a). BAT accumulates an increased amount of circulating succinate, the plasma level of which increases in early arousal (Tb 7–12.8°C) of 13-lined ground squirrels (D’Alessandro et al., 2017). During late arousal (from 18 to 25°C), plasma succinate levels dropped sharply. This suggests that after arousal peripheral tissues supply succinate to BAT through the circulation. Succinate oxidation in mitochondria can lead to increased ROS production (Tretter et al., 2016). Recently, it was shown that activation of succinate dehydrogenase is involved in triggering NST through ROS production in mitochondria (Mills et al., 2018). Indeed, the level of markers of oxidative stress (TBARS and protein carbonyl groups) in BAT of ground squirrels after arousal was significantly higher compared to the torpid state (Orr et al., 2009).

According to our visual observations, during arousal shivering arises at a Tb of 15–18°C, while at a Tb of 25–27°C its intensity reaches maximum values. Almost the same data were obtained during the arousal of a bat (Mejsnar and Janský, 1970). Shivering boosts the metabolic rate and oxygen uptake, thereby promoting considerable ROS production in mitochondria (Nunnally et al., 2011). Our data indicate that during the arousal of ground squirrels, RBCs run through the stage of activation of free radical processes, with the Tb 25°C being critical. At this Tb plasma uric acid levels are maximal (Figure 3A). Previously, an analysis of the uric acid level, which is an indicator of ROS production *in vivo* (Parks et al., 1999; Tøien et al., 2001; Osborne and Hashimoto, 2006) showed that its accumulation in the plasma peaks in the middle phase of arousal: in the Arctic ground squirrels at Tb 10°C (rewarming occurred at an ambient temperature of 21°C; Tøien et al., 2001) and in the Syrian hamsters at Tb 32°C (Okamoto et al., 2006). Moreover, maximum accumulation of uric acid in both rodent species coincided with maximum oxygen uptake. According to the authors, this indicates the existence of systemic oxidative stress at a certain stage of arousal. Considering the literature data, it can be argued that during the arousal of ground squirrels xanthine oxidase in vascular and hepatic endothelial cells is activated at Tb 25°C, due to which the uric acid level and ROS production increase. This could be yet another reason for the development of oxidative stress in the blood. It should also be noted that plasma uric acid may have antioxidant functions. Being a major water-soluble antioxidant in the blood plasma with metal-chelating properties, uric acid (as well as ascorbic acid; Tøien et al., 2001; Osborne and Hashimoto, 2006) can directly scavenge singlet oxygen, ^•^OH and peroxide radicals, as well as the oxidants that form enzymatically (Stocker and Keaney, 2004; Glantzounis et al., 2005). It is supposed (Song et al., 2019) that uric acid can function as a blood antioxidant protecting RBCs from oxidative damage, potentially increasing their life span and function. In mammalian blood vessels, NO produced by endothelial cells is involved in smooth muscle relaxation and vasodilation (Zhao et al., 2015). By controlling the O_2_^•-^ level, which can react with NO, SOD promotes the preservation of the physiological function of NO. In the arousal dynamics, ecto-SOD (Okamoto et al., 2006; Akita et al., 2007), on the one hand, and RBC SOD, on the other hand, maintain the vascular NO level by removing O_2_^•-^ and thus play an important role in maintaining the requisite vascular tone and blood pressure (Fukai, 2002). It is noteworthy that during the arousal of ground squirrels, the plasma NO level steadily increases, however this increase becomes particularly significant upon reaching the Tb 25°C (Figure 3B). Our results are consistent with the data by Avci et al. (2014) who found a high level of stable NO metabolites in the liver, lungs, and heart the aroused Asia Minor ground squirrels (*Spermophilus xanthoprymnus*) compared to torpid animals. During the arousal (Tb 18–25°C) of the 13-lined ground squirrels, the L-arginine plasma level is significantly reduced, probably due to its utilization for NO production (D’Alessandro et al., 2017).

The changes in the NO level, which we found in the arousal dynamics of ground squirrels, are also consistent with the expression level of NO synthase in endothelial cells. Using an electron immunocytochemical approach, Saitongdee et al. (1999) demonstrated that in endothelial cells of the renal and mesenteric arteries of the torpid golden hamsters, the number of cells containing NO synthase was significantly less than in the control, while during late arousal (when the Tb rose from 9 to 32°C within 2h) the count of these cells was practically indistinguishable from that in the control.

It has been shown that during arousal, the anterior part of the animal body rewarms up first due to NST (BAT) and then at the expense of ST (skeletal muscles; Hashimoto et al., 2002). At the onset of arousal, the blood volume in the posterior part of the body (kidneys and hindlimbs) is significantly reduced due to vasoconstriction (Osborne et al., 2005). It is generally believed that vasoconstriction of the peripheral and hindlimb vessels is necessary to separate the warmer blood in the anterior part of the body, which comprises the major organs (heart, brain) and BAT, from the colder blood in the posterior part. In 13-lined ground squirrels, it was established that blood flow velocity in tissues located behind the diaphragm picks up after the temperature of the thoracic region rises to 25°C (Bullard and Funkhauser, 1962). In hedgehogs, during arousal upon reaching a Tb of 20°C, the systemic vascular resistance significantly decreased mainly due to a reduction in blood viscosity in the anterior, warmer, and a part of the body (Kirkebö, 1968). At the same time, in colder tissues located in the posterior part of the body, the blood flow velocity remained significantly lower due to a high blood viscosity.

A significant increase in the plasma NO level that we found after the Tb rose up to 25°C appears to be important for the acceleration of blood flow, which is necessary to rewarm the posterior part of the body. In aging, erythrocytes are well known to undergo vesiculation during which the cell loses a part of its membrane. This leads to a decrease in the surface-to-volume ratio, dehydration, and an increase in the mean corpuscular hemoglobin concentration (MCHC; Bosch et al., 1994; Bosman, 2013). Altogether, this entails reduced membrane elasticity and RBC deformability (Linderkamp et al., 1993; Bosch et al., 1994). Previously, we found a significant increase in the MCHC value and a decrease in the mean corpuscular volume (MCV) in the torpid state and during arousal from hibernation up to Tb 25°C (Shihamirova et al., 2020). After that, the MCHC value decreased while the MCV increased. These data suggest that before Tb had reached 25°C, the deformability of RBC was low.

Free radical damage can have serious consequences for RBCs (Cortese-Krott and Shiva, 2019). Oxidative modifications not only have a direct damaging effect on hemoglobin but also were shown to strongly affect (1) flexibility of the cytoskeleton through the formation of cross-links at spectrin-actin-ankyrin nodes, (2) asymmetry of the cell membrane lipid bilayer with exposure of phosphatidylserine, and (3) general deformability and fragility of cells (Lux, 2016; Kuhn et al., 2017). Deformability is a fundamental property of RBCs, which allows them to pass through the microcirculatory bed. LPO (Kuypers et al., 1990), oxidative modifications of membrane proteins, and reduced cytoskeletal flexibility contribute to a decrease in RBC deformability (Suzuki et al., 2007; Grau et al., 2013). Since at Tb 25°C, the degree of oxidative damage to membrane lipids and proteins increases significantly, it can be assumed that RBC deformability would also be reduced during this period.

Although endothelial cells are major NO producers, other cells circulating in the blood (platelets, monocytes, and erythrocytes) are also involved in its production. It has been shown that RBCs are able to synthesize NO (Kleinbongard, 2006; Cortese-Krott et al., 2012, 2018; Yang et al., 2013). Being produced in erythrocytes, NO can act as an autocrine factor, modulating RBC deformability and promoting RBC passage through capillaries and improving blood flow in microcirculation (Bor-Kucukatay et al., 2005; Ulker et al., 2013).

Recently it was shown that nitric oxide *per se* does not affect RBC deformability but prevents its reduction under conditions of oxidative stress (Diederich et al., 2018). Consequently, a significant increase in the plasma NO level after reaching Tb 25°C must increase RBC deformability and hence improve their functional activity.

It is well known that RBCs are constantly exposed both to endogenous and exogenous ROS (Pandey and Rizvi, 2011; Orrico et al., 2018). The main source of intracellular ROS in RBCs is oxyhemoglobin autooxidation generating superoxide, dismutation of which yields hydrogen peroxide (Kuhn et al., 2017). Our results obtained on LGS indicate an increased ROS influx from the plasma into RBCs and/or an intensification of their production in RBCs themselves with increasing Tb up to 25°C. The reasons that cause an increase in RBC ROS levels leading to oxidative stress still need to be elucidated. As known, superoxide can react directly with NO to form ONOO^−^. At physiological pH values, a direct biradical reaction of NO with O_2_^•-^ proceeds almost thrice as fast as SOD-mediated O_2_^•-^ dismutation (Beckman and Koppenol, 1996). A decrease in SOD activity at Tb 25°C can stimulate peroxynitrite production. ONOO^−^ is a strong oxidizing and nitrating agent towards a wide range of macromolecules. Moreover, it is considered to be relatively stable under physiological conditions and can spread over distances equal to several cell diameters, which makes it much more toxic (Szabó et al., 2007). SOD is the only antiradical enzyme which catalyzes dismutation of the superoxide anion radical into hydrogen peroxide. CAT decomposes H_2_O_2_ into water and O_2_. Antioxidant defense requires a coordinated activity of these enzymes. During arousal, up to reaching Tb 30°C, the SOD/CAT ratio increases linearly (Figure 5D). An imbalance in this ratio indicates the induction of ROS accumulation in RBCs, which peaks at Tb 30°C. A decrease in the activity of SOD and CAT under conditions of oxidative stress can be caused by several reasons. As mentioned above, Cu/Zn-SOD activity can change due to oxidative modification of the free cysteine residue. It has also been established that fatty acid hydroperoxides can oxidize thiols to sulfinic (-SO_2_H) and sulfonic acids (-SO_3_H; Appolinário et al., 2015). In addition to the ability to dismute the superoxide radical, Cu/Zn-SOD also exhibits a peroxidase activity, and this reaction inhibits the enzyme (Hodgson and Fridovich, 1975). It was shown that the interaction of intact RBCs with H_2_O_2_ leads to inactivate endogenous Cu/Zn-SOD in a concentration-dependent manner (Sinet and Garber, 1981; Salo et al., 1988). In this case, SOD inhibition is due to the interaction of H_2_O_2_ with the enzyme’s copper ions, which generates a hydroxyl radical (OH^•^) mediating SOD inactivation (Jewett et al., 1999). It has been demonstrated in some studies that superoxide radicals are potent inhibitors of CAT (Kono and Fridovich, 1982). CAT is a heme-containing protein which binds NO (Deisseroth and Dounce, 1970). *In vitro* experiments have shown that at physiological concentrations, NO reversibly inhibits CAT (Brown, 1995). This may be due to the CAT-mediated NO destruction, as reported to occur in the presence of hydrogen peroxide *in vitro*. Nitric oxide binds to iron in the CAT heme group (Brown, 1995) and competes with hydrogen peroxide for these sites, which can lead to increased accumulation of H_2_O_2_ in cells.

Our data indicate that after the complete arousal of animals, the intensity of free radical processes in RBCs decreases, while the activity of the antioxidant defense components increases. Thus, oxidative stress arising at Tb 25°C is transient. What are the ways of activating the adaptive mechanisms that lead to an increase in the effectiveness of antioxidant defense in RBCs after an elevation of Tb above 25°C?

Our data show that the activity of SOD and CAT increases significantly during the arousal from hibernation. This increase can be associated with the synthesis of new enzyme molecules and their post-translational modifications. In non-nuclear RBCs of ground squirrels, the synthesis of enzymes is not possible, but it can occur in the bone marrow precursors of RBCs, which then enter the blood in the form of reticulocytes. During arousal, the number of reticulocytes in the blood increases from 1.5% (torpid state, Tb 4°C) up to 3.1% after reaching Tb 30°C (Shihamirova et al., 2020). It is unlikely that a slight increase in the level of reticulocytes relative to the total number of RBCs significantly affects SOD and CAT activity in RBCs. It is more likely that during the arousal, SOD and CAT molecules undergo chemical modification by cysteinylation (Auclair et al., 2013), glutathionylation, and phosphorylation (Banks and Andersen, 2019). But for the RBCs enzymes of ground squirrels in the topor-arousal cycle, this needs to be confirmed experimentally.

Oxygen-dependent metabolic modulation in RBCs leads to an increase in glycolytic fluxes in response to low oxygen tension, as well as to activation of the pentose phosphate pathway to counteract oxidative stress arising in response to high oxygen tension (Castagnola et al., 2010). Indeed, high oxygen tension promotes ROS production in the Fenton and Haber-Weiss reactions in iron-rich RBCs (Kuhn et al., 2017). Oxidative stress can promote a partial blockade of glycolysis and switching of metabolic fluxes *via* a pentose phosphate pathway by means of two major mechanisms (Gehrke et al., 2019): (1) binding glycolytic enzymes to the N-terminus of the band 3 protein (the region occupied under hypoxia by deoxyhemoglobin), and (2) oxidizing functional thiol groups in active centers of the rate-limiting glycolytic enzymes, including glyceraldehyde dehydrogenase. Indeed, RBCs of the hibernating ground squirrel are characterized in the torpid state by an increased production of the glycolytic by-products pyruvate and lactate, while during arousal there is a sudden increase in the rate of glucose oxidation through the pentose phosphate pathway (Gehrke et al., 2019) which provides NADPH required for the reduction of GSSG (oxidized glutathione) and MetHb. Such switching of glucose oxidation may have occurred while re-warming ground squirrels after their Tb had reached 25°C, as evidenced in our experiments by an increase in the GSH level in RBCs (Figure 6). A restoration of the oxidative stress-depleted GSH level in RBCs while further increasing Tb above 25°C appears to occur also due to an increase in GSH synthesis or a regeneration of mixed disulfides in proteins. It has been shown that during the arousal of ground squirrels, the pathways leading to accumulate amino acid precursors of glutathione were activated (Gehrke et al., 2019).

## Conclusion

Our results are the first evidence that oxidative stress develops in the erythrocytes of ground squirrels during arousal from hibernation when the body temperature reaches 25°C. In the torpid state (Tb 4°C), the generation of ROS and RNS, as well as the degree of oxidative damage of lipids and proteins of RBCs membranes is at a low level. At the same time, the enzymatic antioxidant protection is significantly reduced. During arousal, the generation of ROS and RNS increases, as well as the degree of oxidative damage of lipids and proteins, reaching a maximum at Tb 25°C. In this case, oxidative stress is accompanied by a decrease in GSH levels, SOD, and CAT activities and their disbalance. However, after full recovery of body temperature, the traces of oxidative stress completely disappeared, which is associated with a significant increase in the activity of the antioxidant defense system of erythrocytes. Our study confirms the hypothesis that oxidative stress, which develops at the late stage of arousal was transient and physiologically regulated.

## Data Availability Statement

The original contributions presented in the study are included in the article/supplementary material, further inquiries can be directed to the corresponding author.

## Ethics Statement

The animal study was reviewed and approved by Bioethics Committee of Sechenov Institute of Evolutionary Physiology and Biochemistry Russian Academy of Sciences.

## Author Contributions

NK, EN, ZS, MA, ShCh, and AK contributed to the design. ZS, MA, and ShCh contributed to the experiments. NK, EN, and AK contributed to the data analysis. NK drafted the article. All authors contributed to the article and approved the submitted version.

## Funding

This work was done in the frame of the State Assignment AAA-A18-118012290371-3, Russia.

## Conflict of Interest

The authors declare that the research was conducted in the absence of any commercial or financial relationships that could be construed as a potential conflict of interest.

## Publisher’s Note

All claims expressed in this article are solely those of the authors and do not necessarily represent those of their affiliated organizations, or those of the publisher, the editors and the reviewers. Any product that may be evaluated in this article, or claim that may be made by its manufacturer, is not guaranteed or endorsed by the publisher.
